# Biological Activity of *Picrorhiza kurroa*: A Source of Potential Antimicrobial Compounds against *Yersinia enterocolitica*

**DOI:** 10.3390/ijms232214090

**Published:** 2022-11-15

**Authors:** Anju Thapa, Ravinder Kaushik, Smriti Arora, Sundeep Jaglan, Varun Jaswal, Virendra Kumar Yadav, Manjeet Singh, Aarti Bains, Prince Chawla, Azhar Khan, Melinda Fogarasi, Szabolcs Fogarasi

**Affiliations:** 1 Faculty of Biotechnology and Allied Sciences, Shoolini University, Solan 173229, Himachal Pradesh, India; 2 School of Health Sciences and Technology, University of Petroleum and Energy Studies, Dehradun 248001, Uttarakhand, India; 3 CSIR-Indian Institute of Integrative Medicine, Jammu 180001, Jammu and Kashmir, India; 4 Department of Biosciences, School of Liberal Arts and Sciences, Mody University of Sciences and Technology, Lakshmangarh, Sikar 332211, Rajasthan, India; 5 Department of Microbiology, Lovely Professional University, Phagwara 144411, Punjab, India; 6 Department of Food Technology and Nutrition, School of Agriculture, Lovely Professional University, Phagwara 144411, Punjab, India; 7 Department of Food Engineering, University of Agricultural Sciences and Veterinary Medicine of Cluj-Napoca, Calea Mănăstur 3–5, RO-400372 Cluj-Napoca, Romania; 8 Department of Chemical Engineering, Faculty of Chemistry and Chemical Engineering, Babeş-Bolyai University, 11 Arany Janos Street, RO-400028 Cluj-Napoca, Romania; 9 Interdisciplinary Research Institute on Bio-Nano-Sciences, Babeş-Bolyai University, 42 Treboniu Laurian Street, RO-400271 Cluj-Napoca, Romania

**Keywords:** *P. kurroa*, antimicrobial potential, picroside–1, *Yersinia enterocolitica*

## Abstract

Yersiniosis, caused by *Yersinia enterocolitica*, is the third most rampant zoonotic disease in Europe; the pathogen shows high antibiotic resistance. Herbs have multiple anti–microbial components that reduce microorganism resistance. Therefore, an extract of *Picrorhiza kurroa* (*P. kurroa*) was evaluated for potential antimicrobial activity. We report that the ethanolic extract of *P. kurroa* showed effective antimicrobial activity (zone of inhibition: 29.8 mm, Minimum inhibitory concentration (MIC): 2.45 mg/mL, minimum bactericidal concentration (MBC): 2.4 mg/mL) against *Yersinia enterocolitica*. Potential bioactive compounds from *P. kurroa* were identified using LC–MS, namely, cerberidol, annonidine A, benzyl formate, picroside–1, and furcatoside A. *P. kurroa* showed effective antimicrobial potential in skim milk at different pH, acidity, and water activity levels. *P. kurroa* affected the physiology of *Yersinia enterocolitica* and reduced the number of live cells. *Yersinia enterocolitica*, when incubated with *P. kurroa* extract, showed lower toxin production. Picroside–1 was isolated and showed higher antimicrobial potential in comparison to the standard antibiotic. Picroside–1 lysed the *Yersinia enterocolitica* cells, as observed under scanning electron microscopy. Docking revealed that picroside–1 (ligand) showed both hydrophilic and hydrophobic interactions with the dihydrofolate reductase (DHFR) protein of *Yersinia enterocolitica* and that DHFR is a possible drug target. The high activity and natural origin of Picroside–1 justify its potential as a possible drug candidate for *Yersinia enterocolitica*.

## 1. Introduction

*Yersinia enterocolitica* is a global food–borne pathogen that causes gastrointestinal diseases (yersiniosis) and non–gastrointestinal infections (pneumonia, meningitis, and epidermic infection) in people and also in livestock, deer, pigs, fish, and birds [1]. 

The etiological factor of yersiniosis is a Gram–negative, rod–shaped, non–spore–forming, common food–borne pathogen that constitutes a group of 6 biogroup strains, with more than 57 O serogroups. It is one of the 17 species comprising the *Yersinia enterocolitica* genus, which belongs to the Enterobacteriaceae family. The most prevalent pathogenic strains fall in the serogroups O:3, O:5, 27, O:8, and O:9. The establishment of the pathogen results in gastrointestinal illnesses that do not require antibiotic therapy [2]. Acute enteritis and terminal ileitis, fever, vomiting, abdominal discomfort, and occasionally bloody and watery diarrhea are the most typical symptoms in cases of severe *Yersinia enterocolitica* infection. There is currently a great deal of interest in plant–derived components as food additives. Several essential oils and plant extracts (*Murraya koenigii*, *Tanacetum vulgare*, and *Tanacetum balsamita*) are investigated against *Yersinia enterocolitica* [3]. 

*P. kurroa* belongs to the family *Scrophulariaceae*; the ethanol and methanol extracts of *P. kurroa* contain vital bioactive compounds that exert potential antibacterial activity. It has been reported that the ethanolic extract of *P. kurroa* inhibits the growth of several pathogenic microorganisms, including *Escherichia coli*, *Staphylococcus aureus*, *Streptococcus pyrogens*, *Salmonella typhi*, and *Klebsiella pneumoniae*, whereas the methanolic extract kills *Pseudomonas aeruginosa* and *Staphylococcus aureus*, respectively [4]. In addition, picroside–1 is the major chemical constituent present in *P. kurroa*, and this compound is used in the preparation of herbal drugs. The medicinal importance of *P. kurroa* is very high, due to its being a rich source of picroside–1 and picroside–11 [5,6]. In this context, Jannathul et al. [7] analyzed the methanolic and aqueous extracts of *P. kurroa* and reported their potential antibacterial and antifungal activities, respectively. Rathee et al. (2012) [5] prepared methanolic and ethanolic extracts of *P. kurroa*, and reported that the methanolic extract showed significant antimicrobial activity against *Staphylococcus aureus* and *Pseudomonas aeruginosa*. Ganeshkumar et al. (2013) [8] also showed that the methanolic extract of *P. kurroa* exhibited significant antibacterial activity when compared with ciprofloxacin, a standard drug, using the cup–plate method. Similarly, Ghansar et al. (2012) [8] showed the antimicrobial activity of *P. kurroa* against *Staphylococcus aureus* and *Candida albicans*, while Shaba et al. (2015) [9] showed that *P. kurroa* has anti–trypanosomal activity against *Trypanosoma evansi*. Nisar et al. (2022) [10] and Mehta et al. (2021) [11] reported that *P. kurroa* is rich in alkaloids, tannins, steroids, flavonoids, saponins, and phenolics. It has also been observed that this is a medicinal herb found in the Himalayan region on a large scale; several studies have revealed antimicrobial activity when using it against the human pathogens of *Pseudomonas aeruginosa*, *Escherichia coli* (MTCC1697), *Staphylococcus aureus* (MTCC96), and *Salmonella typhimurium* (MTCC98) [12]. In addition, Dheer et al. (2021) [13] observed that the methanol extract of *P. kurroa* has antimicrobial potential against *Escherichia coli*. The above findings showed that *P. kurroa* extract offers potential antimicrobial activity; therefore, in the present study, we selected the extract of *P. kurroa* to identify the antimicrobial activity against *Yersinia enterocolitica* using different standard protocols and assays, including the agar–well diffusion method, MIC, and MBC. Liquid chromatography–mass spectroscopy (LC–MS) was used to analyze the compounds. Microscopic observation of the effect of antibacterial compounds on the physiology of the microorganism was analyzed using a scanning electron microscope. Chemical constituents from the herbs were isolated using column chromatography and characterization using nuclear magnetic resonance (NMR). The effect of the antibacterial compounds on the dihydrofolate reductase (DHFR) protein of *Yersinia enterocolitica* was analyzed using docking.

## 2. Results

### 2.1. The Antimicrobial Potential of the Aqueous, Petroleum Ether, and Ethanolic Extract of P. kurroa against Yersinia enterocolitica, Using Agar–Well Diffusion, MIC, and MBC

The ethanolic extract of *P. kurroa* showed a significantly (*p* < 0.05) higher zone of inhibition (ZOI) in comparison to the aqueous and petroleum ether extracts (Table 1). 

The ethanolic extract of *P. kurroa* showed the highest ZOI compared to the standard antibiotic, ciprofloxacin. The aqueous and petroleum ether extracts of herbs showed lower antimicrobial potential in comparison to ciprofloxacin. The activity index of ethanolic extract was higher in comparison to aqueous and petroleum ether extracts (Table 1). The MIC and MBC of *P. kurroa* were determined and the results are presented in Table 1. In the case of MIC, ethanolic extract showed the lowest MIC in comparison to the aqueous and petroleum ether extracts. Based on the antimicrobial results of MIC and MBC, the ethanolic extract of *P. kurroa* showed the highest antimicrobial potential; therefore, this was selected for further study. *P. kurroa* has been widely studied for antimicrobial activity and several studies reported that it possessed significant antimicrobial activity; however, its effect on *Yersinia enterocolitica*, the causative agent of yersiniosis, has not been studied until now. 

### 2.2. Antimicrobial Potential of Ethanolic Extracts of the P. kurroa Rhizome, Checked in Skim Milk, at Different pH, Acidity, and a_w_

Microorganisms have different optimal conditions for growth, such as nutrients, pH, acidity, and water activity [14]. The general trend is that as pH, a_w_, and nutrients decrease, the acidity of the media increases; a decrease in microbial growth is observed. Milk is a rich medium for microbial growth [10]. As oral drugs are ingested and mixed with food in the digestive system, we therefore checked the effect of *P. kurroa* in milk (Table 2). 

After 12 and 24 h incubation, *P. kurroa* showed no bacterial growth in skim milk. The ethanolic extract of the *P. kurroa* rhizome reduced the number of bacterial cells significantly in comparison to the control (ciprofloxacin). The *P. kurroa* rhizome showed significant antimicrobial potential in the presence of food, compared to antibiotics. This reflects its importance as an antimicrobial agent. The antimicrobial potential of the ethanolic extract of *P. kurroa* at the different pHs of 3, 4, 5, and 6 was determined (Table 2). The viable cell count was determined after the use of different pH solutions for up to 10^−7^ dilutions. At the different pHs of 3, 4, 5, and 6, *P. kurroa* showed no bacterial growth. The antimicrobial potential of the ethanolic extract of the *P. kurroa* rhizome under different acidic conditions was determined; the results are presented in Table 2. Out of all the acidity concentrations (HCl), *P. kurroa* showed the highest zone of inhibition in comparison to the control (ciprofloxacin). From the above results, it is clear that as the acidity increases, the antimicrobial potential increases. The antimicrobial potential of the ethanolic extract of the *P. kurroa* rhizome against *Yersinia enterocolitica* was determined at a different level of water activity (a_w_) and the results are presented in Table 2. In the case of water activity, *P. kurroa* showed the highest zone of inhibition and higher antimicrobial potential in comparison to the control (ciprofloxacin). The results revealed that as the water activity decreases, the antimicrobial potential of *P. kurroa* increases. At present, no study that checked the effects of pH, food source, acidity, and water activity on *Yersinia enterocolitica* in the presence of *P. kurroa* has been made. However, our results follow a general trend. From the above results, it was clear that *P. kurroa* was effective in a wide pH range, as well as acidity, water activity, and the food system. The antimicrobial potential of herbal extracts was assessed by the researchers against microorganisms at different pH levels (pH 3, 4, 5, and 6), at different acidity levels (0.4%, 2%, and 4%), and water activity levels (0.99 a_w_, 0.95 a_w_, 0.85 a_w_, and 0.75 a_w_). However, no study was carried out on the antimicrobial potential of the ethanolic extract of *P. kurroa* against *Yersinia enterocolitica*.

### 2.3. Microscopic Observation of the Effect of the Herbal Extract of the P. kurroa Rhizome on the Physiology of the Microbe

**Figure 1:**
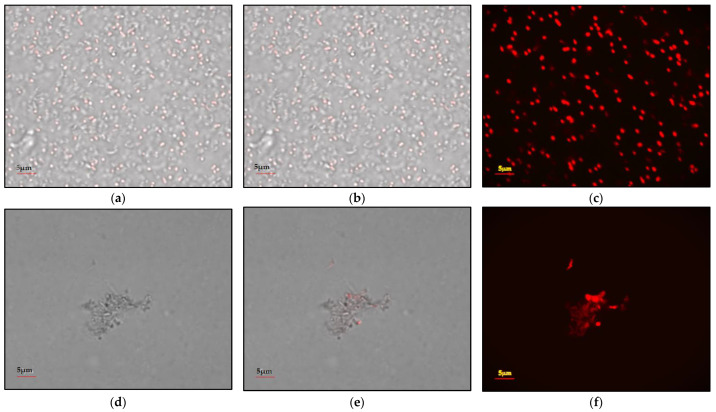
Effect of the herb extract on *Yersinia enterocolitica*, analyzed using confocal microscopy. (**a**–**c**) Control sample (*Yersinia enterocolitica*); (**d**–**f**) *Yersinia enterocolitica* sample, treated with the ethanolic extract of *P. kurroa*.

The microscopic examination of microorganisms is the gold standard by which to determine the physiology of microorganisms [15]. The effect of the *P. kurroa* extract on the physiology of *Yersinia enterocolitica* was observed using a confocal microscope. By this means, the two–dimensional (2D) structure of the sample was observed at 529 nm. *Yersinia enterocolitica* was treated with propidium iodide dye for 30 min and propidium dye penetrated the dead cells, which were stained pink in color. However, the dye did not penetrate the live cells, which remained colorless [16]. The control sample (without herbal extract) and bacterial strain treated separately with *P. kurroa* have three images for identification, based on the physiology and the dead and living cells. Figure 1a shows the total number of bacterial cells, without discriminating between the dead and living cells. Figure 1b shows the total number of living and dead cells separately, while Figure 1c shows the total number of dead cells. In Figure 1d–f, *Yersinia enterocolitica* cells that were treated with the ethanolic extract of *P. kurroa* showed a lesser number of living and dead bacterial cells; it was also observed that the herbal extracts changed the physiology of *Yersinia enterocolitica*. 

Previously, the effect of herbal compounds on the physiology of microorganisms has been studied using a confocal microscope, however, no study was carried out for the antimicrobial effect of ethanolic extract of *P. kurroa* against *Yersinia enterocolitica*.

### 2.4. Isolation and Characterization of Antibacterial Compounds from P. kurroa

**Figure 2:**
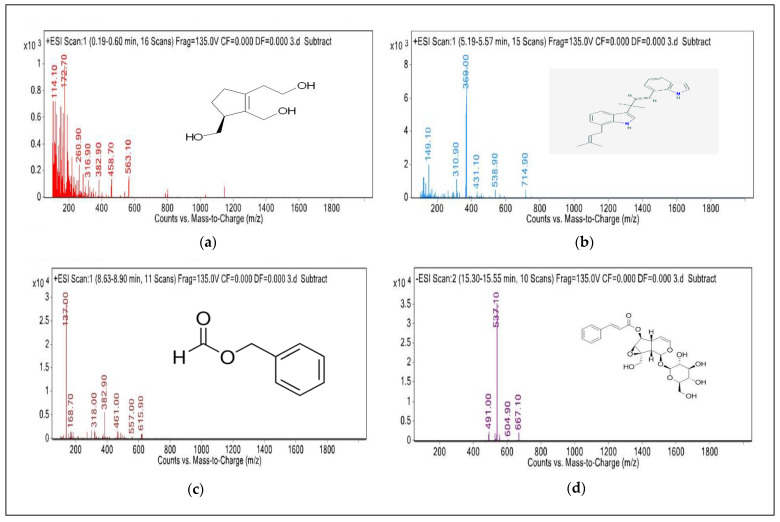

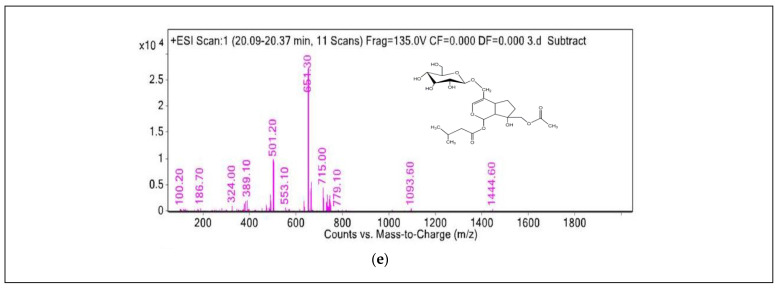
The compounds identified using LC–MS from the ethanolic extract of *Picrorhiza kurroa*: (**a**) cerberidol, (**b**) annonidine A, (**c**) benzyl formate, (**d**) picroside 1, (**e**) furcatoside A.

Five compounds from the *P. kurroa* rhizome were identified using LC–MS (Figure 2). 

The molecular peak (Rt 0.47 min) was identified as cerberidol, with a molecular weight, *m*/*z*, of 172.70 and an empirical formula of C_9_H_16_O_3_. The second peak (Rt 5.36 min) was identified as annonidine A, with a molecular weight, *m*/*z*, of 369.00 and an empirical formula of C_26_H_28_N_2_. The third peak (Rt 8.77 min) was identified as benzyl formate, with a molecular weight, *m*/*z*, of 137.00 and an empirical formula of C_8_H_8_O_2_. The fourth molecular peak (Rt 15.35 min) was identified as Picroside–1, with a molecular weight, *m*/*z*, of 491.00 and an empirical formula of C_24_H_28_O_11_. The fifth peak (Rt 20.24 min) was identified as furcatoside A, with a molecular weight, *m*/*z*, of 651.30 and an empirical formula of C_32_H_42_O_14_. 

### 2.5. Isolation of Chemical Constituents from the P. kurroa Rhizome Using Column Chromatography

**Figure 3:**
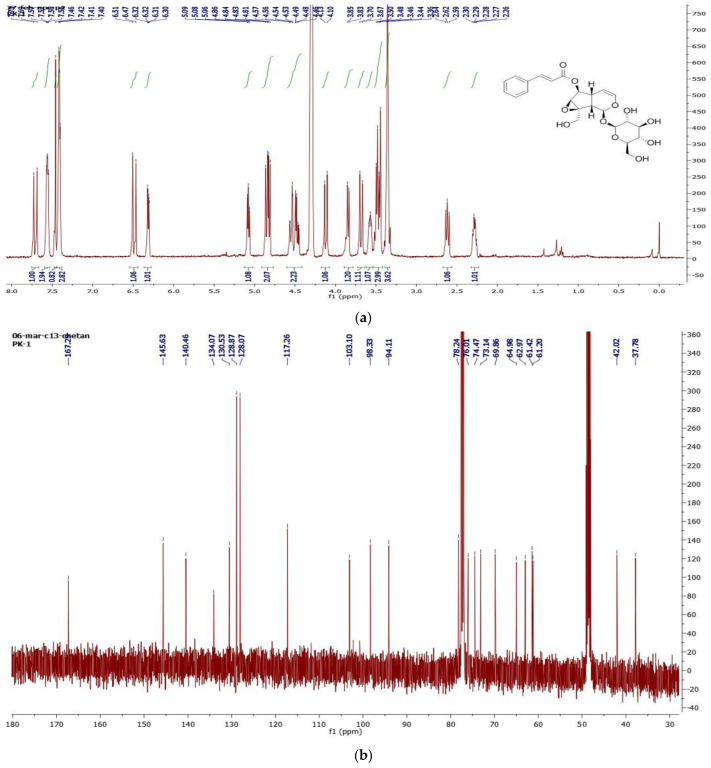
(**a**) The ^1^H NMR and (**b**) ^13^C NMR spectra of picroside–1.

**Figure 4:**
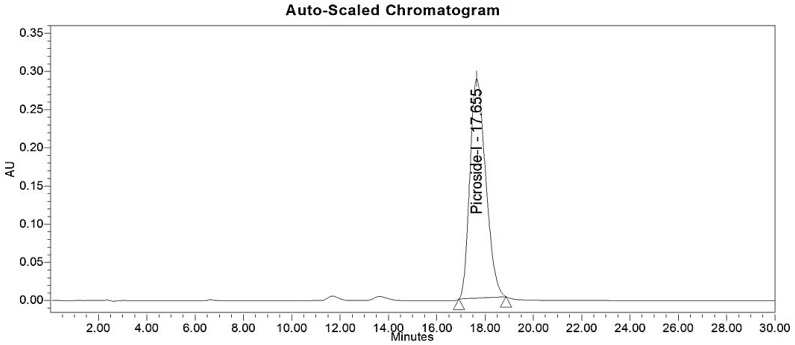
**The** HPLC spectra of picroside–1.

With the use of column chromatography, the compounds present in *P. kurroa* rhizome extract were fractionated, then one compound was isolated and identified using NMR (Figure 3). The isolated compound was found to be picroside–1. The purity of picroside–1 was checked using RP–HPLC and was found to be 98% pure. Picroside–1 has a grayish–brown color, with a molecular weight of 492.47. The molecular formula of picroside–1 is C_24_H_28_O_11_; the melting point of picroside–1 is 128–130 °C. In line with the NMR analysis, the CDCl_3_ was used for sample preparation at 1 mg/mL. To summarize: ^1^H NMR (CDCl_3_, 400 MHz) 2.28 (m, H–5), 2.62 (dd, J = 10.8, 8.0, H–9), ^13^C NMR (CDCL_3_, 400 MHz): 167.3 (CO), 145.6 (C–1), 140.5 (C–3), 134.0 (C–1″), 130.5 (C–4″), 128.8 (C–2″and C–6″), 128.0 (C–3″ and C–5″), 117.2, 103 (C–4), 98.3 (C–1′), 94.1 (C–1), 78.2 (C–6), 76.0 (C–3′), 74.4 (C–5′), 73.1 (C–2′), 69.8 (C–4′), 64.9 (C–8), 62.9 (C–6′), 61.4 (C–7), 61.2 (C–10), 42.0 (C–9), 37.8 (C–5). The RP–HPLC was used to check the purity of picroside–1. The sample was prepared in methanol at 1 mg/mL. The best resolution was achieved using a mobile phase consisting of MeOH: water (40:60). The picroside–1 of the methanolic extract of *P. kurroa* showed the peak of purity at a retention time of 17.655 min (Figure 4). Wang et al. (2013) [17] studied the methanolic extract of *P. kurroa* using column chromatography. According to the NMR analysis of picroside–1, DMSO–d6 solvent was used for the sample preparation, i.e., 1 mg/mL for ^1^H NMR and ^13^C NMR. The isolated picroside–1 was evaluated for antimicrobial activity against *Yersinia enterocolitica* because picroside–1 is the major ingredient of *P. kurroa* [8] and is also the most widely researched [18].

### 2.6. Antibacterial Potential of the Ethanolic Extract of Picroside–1

The antimicrobial potential of the ethanolic extract of picroside–1 was determined using the agar–well diffusion method. The picroside–1 compound showed a higher zone of inhibition than ciprofloxacin (control). The activity index of picroside–1 was higher than that of the control (Table 3). 

These results were correlated with earlier studies conducted on the antimicrobial potential of picroside–1 against several microbes, including *Yersinia enterocolitica*, *Bacillus subtilis*, and *Staphylococcus aureus* [6]. The antimicrobial potential of herbal extracts was checked by the researchers against microorganisms; however, no study has until now been carried out for the antimicrobial potential of compounds isolated from herbs against *Yersinia enterocolitica,* using the agar–well diffusion method.

### 2.7. Microscopic Observation of the Effect of Picroside–1 on the Physiology of Yersinia enterocolitica

**Figure 5:**
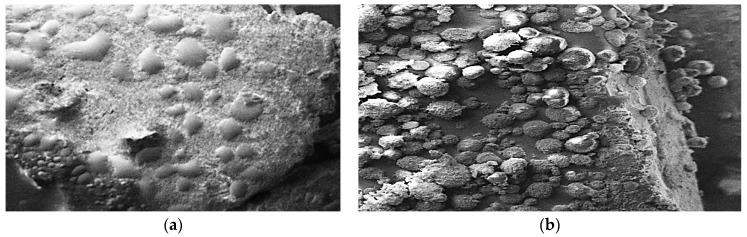
The scanning electron microscope (SEM) of Picroside–1 and *Yersinia enterocolitica*: (**a**) *Yersinia enterocolitica* (control), (**b**) Picroside–1 + *Yersinia enterocolitica*.

The antimicrobial potential of picroside–1 on the physiology of *Yersinia enterocolitica* was observed using a scanning electron microscope. After the addition of picroside–1, the bacterial cells were lysed and showed reduced multiplication of bacteria, in comparison to the control. From the SEM images in Figure 5, it is clear that picroside–1 inhibits cell multiplication and lyses *Yersinia enterocolitica*. Picroside–1 also reduces the microbial count, ruptures the cell wall, and induces cell lysis.

The antimicrobial potential of several herbal extracts has been checked by the researchers against microorganisms using a scanning electron microscope; however, no study has been carried out for the antimicrobial potential of compounds isolated from herbal extracts against *Yersinia enterocolitica,* respectively.

### 2.8. Ramachandran Plots for the Homology Modeling of Dihydrofolate Reductase (DHFR) of Yersinia enterocolitica

The DHFR is an enzyme that is essential for several pathways and the synthesis of the de novo purine, glycine, and the DNA precursor. It is involved in the biosynthesis of tetrahydrofolate, which is part of co–factor biosynthesis. It also plays a role in NADP binding, along with many other functions. All these functions make DHFR an important enzyme in bacterial cell metabolism, cell survival, and growth. Feldman et al. (2002) [19] used DHFR as a model enzyme for the homology modeling of *Yersinia enterocolitica*. Therefore, DHFR was selected as a drug target to verify the effect of picroside–1. The homology–modeled structure of DHFR was validated via a Ramachandran plot, using PROCHEK. (Table 4). The Ramachandran plot analysis for DHFR and *Yersinia enterocolitica* showed that 95.5% of amino acids fell into in the most favored region, 4.5% of amino acids fell into the additionally allowed region, and no amino acids fell into the generously allowed regions and disallowed regions. 

**Figure 6:**
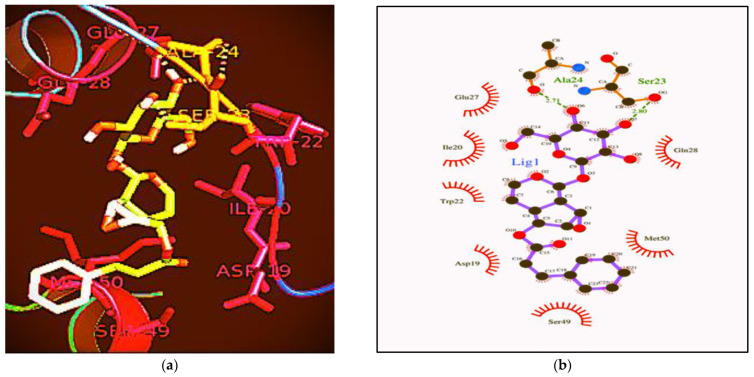
The antimicrobial potential of Picroside–1 on the DfrA1 DHFR protein sequence of *Yersinia enterocolitica*. (**a**) Docking of Picrosode–1. (**b**) Lig–plot of Picroside–1.

According to the parameter quality of the model, the modeling of the DHFR of *Yersinia enterocolitica* was considered to be good (Figure 6—Ramachandran PROCHEK 2018). 

### 2.9. Effect of Picroside–1 on DHFR Protein of Yersinia enterocolitica

The effect of Picroside–1 on the DHFR protein of *Yersinia enterocolitica* was analyzed via a docking study (Table 5). 

The crystal structure of the DHFR protein of *Yersinia enterocolitica* and compound, i.e., Picroside–1, was docked at its active site, using a standard docking protocol. After the docking analysis, it was observed that *Yersinia enterocolitica* DHFR and picroside–I both have hydrophilic and hydrophobic interactions at different sites, namely, 303.24. Ser–23 and Ala–24, which showed hydrophilic interactions between the ligand and protein, whereas Asp–19, Ile–20, Trp–22, Glu–27, Gln–28, Ser–49, and Met–50 showed hydrophobic interactions between the ligand and protein (Figure 6a,b).

## 3. Discussion

*P. kurroa* is an underutilized herb that is rich in phytochemicals and has pharmacological and antimicrobial effects. Indeed, the chemical constituents of the genus Picrorhiza and its crude extracts have antimicrobial effects [20]. *P. kurroa* possesses broad antimicrobial activity, as the zone of inhibition was observed for both Gram–positive and Gram–negative bacterial strains [21]. The antimicrobial activity of the different solvent extracts of *P. kurroa* was analyzed; the ethanolic extract of *P. kurroa* showed a higher antibacterial action against many bacteria [22,23,24]; however, no such study was reported against *Yersinia enterocolitica*. Kumar et al. (2010) [4] reported that the methanol, ethanol, aqueous, hexane, and acetone extracts of the rhizome of *P. kurroa* showed antibacterial activity against Gram–negative and Gram–positive bacteria. Kumar et al. (2010) [4] showed that the aqueous extract of *P. kurroa* showed the lowest MBC and MIC values against *Escherichia coli* (6.05 mm), whereas methanolic extracts have the lowest MBC and MIC values against *Streptococcus pyogenes* (6.06 mm), in comparison to acetone, ethanol, and hexane extracts, respectively. 

The intrinsic and extrinsic factors affected microbial growth in food systems, in terms of nutrient contents, pH, water activity, etc. [25]. Every food has a different chemical composition, pH and acidity range, and water activity. Therefore, it is important to determine the antimicrobial effect of *P. kurroa* in food systems, at different pH and acidity, and water activity on the growth of *Yersinia enterocolitica.* Milk and processed milk products are considered as a complete food and are a key source of macro and micronutrients; therefore, the antimicrobial activity of *P. kurroa* was assayed in milk and significant antimicrobial activity was observed. Schiemann (1980) [26] reported that *Yersinia enterocolitica* grew best at a pH of 7.6 to 7.8 and at 32 °C, with a generation time of 33–39 min. The growth of *Yersinia enterocolitica* at 3, 4, 5, and 6 pH was analyzed; it was observed that with a decrease in pH, the growth of *Yersinia enterocolitica* decreases. It is a universal fact that a decrease in water activity decreases the growth of microorganisms, and a similar effect was also observed for *Yersinia enterocolitica*. Similar results were reported by Donato et al. (2022) [27], who tested the effect of water activity on microorganisms and reported that the growth rate and toxin production was at a maximum at 0.99 a_w_ and, with a decrease in water activity, the growth rate and toxin production were also decreased.

Confocal microscopic studies confirmed that *P. kurroa* has bactericidal and bacteriostatic potential against *Yersinia enterocolitica*. With application of *P. kurroa*, the number of cells decreased, showing that the replication of *Yersinia enterocolitica* was reduced. Most of the cells observed after *P. kurroa* treatment were dead. 

Patil et al. (2013) [28] carried out an LC–MS analysis of a crude extract of the rhizome of *P. kurroa* and reported the presence of picroside–1 and picroside–II, whereas Vipul et al. (2005) [29] carried out an LC–MS analysis and reported the presence of picroside–1 and kutkoside. Sultan et al. (2016) [18] reported that iridoid glycosides, picrosides–I and II, and kutkoside are the principal bioactive molecules. Tabassam et al. (2020) [20] reported that *P. kurroa* also contains Dihydromikanolide and 1,3-Dicyclohexyl-4-(cyclohexylimino)-2-(cyclohexylethylamino)-3,4-dihydro-1,3-diazetium.

## 4. Materials and Methods

### 4.1. Materials

The *P. kurroa* rhizome (Voucher SU19–202BT, age = 2 years) was collected from the local market in Jammu (Jammu and Kashmir, India) and verified by the Department of Botany, Shoolini University, Solan, Himachal Pradesh, India. The *Yersinia enterocolitica* preservation medium was 10% glycerol, and the strain designation code is C975. The growth condition of the bacteria was aerobic and was achieved using a nutrient agar medium for 24 h at 30 °C. The subculturing period for bacteria is 30 days. The MTCC4858 is an accession number of *Yersinia enterocolitica* and was obtained from the IMTECH (Institute of Microbial Technology), Chandigarh. HiMedia chemicals and media of analytical reagent grade were used for the analysis of samples. Ethanol, petroleum ether, dimethyl sulfoxide (DMSO), and the solvents used for gas chromatography–mass spectrometry and high–pressure liquid chromatography were of chromatography grade.

### 4.2. Processing of Herbs and the Preparation of Herb Extracts

*P. kurroa* rhizomes were cleaned and washed with potable water, followed by washing with distilled water, and were stored for drying in a shaded place. The dried herb was powdered, weighed, and stored in a clean container. The extracts of the herbs were extracted using three solvents, namely, aqueous, ethanol, and petroleum ether solvents. The three solvents, each with different polarity, were used to extract the antimicrobial compounds, namely, water (hydrophilic), ethanol (amphophilic), and petroleum ether (hydrophobic). The extraction was carried out using the Soxhlet apparatus for 12 h to 14 h at 50 °C. The herbal extracts were dried in a ceramic dish at 60 °C on a water bath by evaporation and stored at 4 °C in an air–tight container. Then, 5 g of herb powder was added with 50 mL ethanol, petroleum ether, and water, respectively. The yield of the aqueous extract was 21.1%, the ethanolic extract was 20.3%, and the petroleum ether extract was 15.8% [13].

### 4.3. Antimicrobial Assay

The effect of *P. kurroa* extracts on *Yersinia enterocolitica* was determined using the agar–well diffusion assay [30]. The antimicrobial potential was measured as the zone of inhibition, using a metric–scale ruler, and the results were reported in millimeters (mm). The antimicrobial potential of *P. kurroa* rhizome extracts was also assessed by determining the MIC and MBC [15]. The MIC value was determined using various concentrations of stock 15, 7.5, 6.25, 3.75, 1.875, 0.937, 0.468, and 0.234 mg/mL, assayed against the test pathogen using a spectrophotometer. The dilution yielded no visible microbial colony in the MBC using the spread–plate method.

### 4.4. Effectiveness of P. kurroa against Yersinia enterocolitica in the Food System

The antimicrobial potential of the *P. kurroa* rhizome in skim milk was determined by the serial dilution agar plate technique, as described by Najda et al. (2021) [31]. The *Yersinia enterocolitica* bacterial culture was inoculated into Muller–Hinton broth. The incubation temperature was 37 °C for 18 h. The number of microorganisms was adjusted to 1 × 10^7^ CFU/mL (standardized using the 0.5 McFarland standard) and microbial colonies were transferred into normal saline (NaCl). Next, 1 g of skim milk powder was transferred to the test tubes containing sterilized 9 mL of double–distilled water (control). A control experiment was performed, with 1 mL of bacterial culture added to a test tube containing 8 mL of skim milk and 1 mL of 20 ppm ciprofloxacin (reference). In the test experiment, 1 mL of bacterial culture and 100 mg of ethanolic herbal extract were transferred to the test tubes, containing 8 mL of skim milk (sample), and incubated at 37 °C for 24 h. Serial dilution was carried out up to 10^−7^ dilutions, then 1 mL of suspension from 10^−7^ dilutions was spread using a sterile spreader over the entire surface of a deoxycholate citrate agar plate to obtain colonies. The plates were incubated at 37 °C for 12 to 24 h for bacterial growth, while bacterial colonies were counted using the Quebec colony counter (Richerd Darkfield, New York, NY, USA).

#### 4.4.1. Effect of pH

The antimicrobial potential of an ethanolic extract of the *P. kurroa* rhizome was tested at four different pH levels, namely, 6, 5, 4, and 3, determined by viable cell counts as described by Najda et al. (2021) [31]. The *Yersinia enterocolitica* culture was inoculated into Muller–Hinton broth and incubated at 37 °C for 18 h. The number of microorganisms was adjusted to 1 × 10^7^ CFU/mL (standardized using the 0.5 McFarland standard) and the adjusted inoculum was transferred into normal saline. Bacterial culture (1 mL) was added to test tubes with different pHs, i.e., 3, 4, 5, and 6. In addition, 1 mL of bacterial culture and 100mg of ethanolic herbal extract were added to the test tubes containing 8 mL of Muller–Hinton broth, incubated for 24 h at 37 °C. Serial dilution was carried out up to 10^−6^ dilution and 1 mL of suspension from 10^−6^ dilution was spread over the surface, using a sterile spreader on the deoxycholate citrate agar plate. The plates were incubated for 12 and 24 h at 37 °C for bacterial growth. The counting of bacterial colonies was carried out using the Quebec colony counter and the growth was compared with that in the control. 

#### 4.4.2. Effect of Water Activity on Antimicrobial Potential

The antimicrobial potential of *P. kurroa* rhizome at different percentages of normal saline i.e., 0.99, 0.95, 0.85, and 0.75 water activity (a_w_) was determined by the above–mentioned agar–well diffusion assay. 

#### 4.4.3. Effect of Acidity on Antimicrobial Potential

The antimicrobial potential of *P. kurroa* rhizome at different acidities, i.e., 0.04%, 2%, and 4% (adjusted using HCl), were determined by the above–mentioned agar–well diffusion assay.

### 4.5. Microscopic Observation of the Effect of P. kurroa on the Physiology of Yersinia enterocolitica

Confocal microscopy of the bacteria was carried out using the method described by Stiefel et al. (2015) [32]. *Yersinia enterocolitica* was grown in Muller–Hinton broth with 100 mg of ethanolic herbal extract from *P. kurroa*. The 12–well microtiter plates were incubated for 24 h at 37 °C. After incubation, the bacterial culture was washed with phosphate buffer saline to remove the unattached cells. The bacterial strain was treated with propidium iodide dye for 30 min, then, the dye penetrated the dead cells, which were thereby stained pink in color. Microscopic images were clicked using a confocal scanning laser microscope at 592 nm (Fluoview Fv200, Melville, NY, USA). The bacterial smear was approximately 36 to 40 μm thick. The three–dimensional (3D) structure of each sample was obtained using the PLAPDN 60 × 142 objective, with an additional zoom of ×3. The images of the control and sample were compared.

### 4.6. Bioactive Compounds Identification from P. kurroa Using LC–MS

LC–MS, with an electrospray ionization (ESI) interface, was used to determine the bioactive compounds in *P. kurroa*. The LC–MS analysis of the *P. kurroa* rhizome was performed using the method described by [32]. LC–MS analysis was performed on an Agilent 1260 High–Performance Liquid Chromatography Infinity (Agilent Technologies, Santa Clara, CA, USA) series system, attached to an ESI interface and mass spectroscopy detector (Agilent 6410 BQQQ triple–stage quadrupole mass spectrometer). Analytical chromatographic separations of the sample extract were carried out via a chromolith performance RP–18e column (4.6 × 100 mm), protected by a chromolith guard column (Merck, Kenilworth, NJ, USA). The column temperature was maintained at 30 °C and analysis was carried out at a wavelength of 259 nm, with a flow rate of 0.6 mL min^−1^ and a sample injection volume of 7 μL. The following conditions were used throughout the MS experiment: for electrospray ionization with positive ion polarity, the capillary voltage was set to 3 kV, the capillary temperature was 300 °C, the nebulizer pressure was 36 psi, and the drying gas flow rate was 12 L min^−1^.

### 4.7. Isolation of Chemical Constituents from the P. kurroa Rhizome Using Column Chromatography

The antibacterial compound was isolated from the *P. kurroa* rhizome using column chromatography, as described by Nandam et al. (2012) [33]. Methanol was used for sample preparation at 1 mg/mL. The characterization of the isolated compound was analyzed, using NMR to count the number of ^1^H, and ^13^C to finalize the structure of the compound (Bruker AM–500 spectrometer), as described by Lee et al. (2004) [34]. The purification of the compound was analyzed using GC–MS (Perkin Elmer mass spectrometer (Model Claurus 500), as described by Najda et al. (2021) [15]. 

### 4.8. Antibacterial Potential of Picroside–1 against Yersinia enterocolitica

*P. kurroa* showed significant antimicrobial activity against *Yersinia enterocolitica*. The herb’s antimicrobial potential is mainly due to its bioactive compounds. The isolated picroside–1 was analyzed for antibacterial potential and was determined using an agar well–diffusion assay (as per Section 2.3).

### 4.9. Microscopic Observation of the Effect of Antibacterial Compounds on the Physiology of the Microbe

The antimicrobial potential of picroside–1 on the physiology of microorganisms was determined using a scanning electron microscope (Carl Zeiss, EVO 18 Research, Special Edition), as described by Yossa et al. (2014) [35].

### 4.10. Homology Modeling of Dihydrofolate Reductase (DHFR)

DHFR is an important drug target against bacterial as well as other infectious diseases. It reduces the storage ability of tetrahydrofolates in cells, which quality has prime importance for cell survival and multiplication [21]. DHFR is an enzyme that helps in nucleic acid synthesis [35,36]. The crystal structure of DHFR *Yersinia enterocolitica* was not available; therefore, a homology modeling of *Yersinia enterocolitica* was carried out. The template structure was selected through the BLAST PDB database. The best BLAST hit was PDB id 5ecc (*Yersinia enterocolitica*) which has the highest query coverage and percentage identity. Modeler 9.7v (Ben Webb, Department of Biopharmaceutical Sciences and California Institute for Quantitative Biomedical Research, University of California, San Francisco, CA, USA) was used for the homology modeling. Finally, the quality of the structure was studied using the Ramachandran plot.

### 4.11. Docking Studies of the Antimicrobial Compound against Dihydrofolate Reductase (DHFR)

The effect of picroside–1 on the DHFR protein of microorganisms (*Yersinia enterocolitica*) was analyzed via a docking study [37]. The structure of picroside–1 was taken from the PubChem database, and further ligand preparation was carried out using the Autodock tool kit. The docking study was performed using the modeled structure of the DHFR protein. Protein preparation was performed using the AutoDock tool kit; finally, the docking simulation was carried out using AutoDock 4.2, while the 3D structural visualization of the ligand and receptor was analyzed using UCSF Chimera. The 2D interaction and structural visualization of the ligand and receptor were analyzed using Lig Plot+ V.1.4.

### 4.12. Data Analysis

The results of each experiment were documented and analyzed in Microsoft Excel 2007 (Microsoft Corp., Redmond, WA, USA). The results were analyzed to form a single–way analysis of variance (ANOVA) to calculate the critical difference. The means of triplicate readings were determined, along with the SEM (standard error mean) and linear regression analysis, at 95% confidence intervals [38].

## 5. Conclusions

The antimicrobial potential of the ethanolic extract of *P. kurroa* and its bioactive compound, picroside–1, for use against *Yersinia enterocolitica* was investigated. In silico studies on the DHFR of *Yersinia enterocolitica* were also carried out to explore the ligand–protein interaction. The high activity of picroside–1 in comparison to the known drug ciprofloxacin justifies its potential as a drug candidate for *Yersinia enterocolitica*. Therefore, *P. kurroa* can be recommended as a herbal drug to be used for the treatment of yersiniosis.

## Figures and Tables

**Table 1:** Antimicrobial activity of the herbal extract against *Yersinia enterocolitica*, using the agar–well method.

Antimicrobial Substance	Zone of Inhibition (mm)			Activity Index (In Comparison to Ciprofloxacin)		
	Aqueous	Ethanol	Petroleum Ether	Aqueous	Ethanol	Petroleum Ether
Ciprofloxacin	28.4 ± 0.28 ^aA^	29.4 ± 0.23 ^aB^	29.3 ± 0.51 ^aB^	-	-	-
*Picrorhiza kurroa*	21.3 ± 0.34 ^bB^	29.8 ± 0.40 ^aC^	6.5 ± 034 ^bA^	0.75	1.01	0.21
**MIC (mg/mL)**						
*Picrorhiza kurroa*	2.6 ± 0.04 ^cB^	2.45 ± 0.04 ^bA^	2.85 ± 0.05 ^cC^	1.17	0.83	0.90
**MBC (mg/mL)**						
*Picrorhiza kurroa*	2.62 ± 0.02 ^cB^	2.4 ± 0.02 ^bA^	2.82 ± 0.03 ^cC^	-	-	-

The means of triplicate readings were determined, ± SD (standard deviation). Values having different superscripts (^a^, ^b^ and ^c^) in a column showed a significant (*p* < 0.05) difference. Values having different superscripts (^A^, ^B^ and ^C^) in a row showed a significant (*p* < 0.05) difference. MIC: minimum inhibitory concentration; MBC: minimum bactericidal concentration.

**Table 2:** The antimicrobial activity of *P. kurroa* against *Yersinia enterocolitica* in skim milk at different pH, acidity, and a_w_ levels.

Antimicrobial Potential of Herbal Extracts in Milk				
Samples		On 12th h		On 24th h
Milk with Bacterial culture		50 ± 0.05 ^a^		56 ± 0.18 ^c^
Milk with Ciprofloxacin		37 ± 0.57 ^b^		43 ± 1.15 ^b^
Milk with *Picrorhiza kurroa*		0 ± 0.00 ^a^		0 ± 0.00 ^a^
**Antimicrobial Potential of Herbal Extracts at Different pH**				
**Samples**	**Microbial Count (×10^−7^ cfu/mL)**			
	**pH 3**	**pH 4**	**pH 5**	**pH 6**
Ciprofloxacin	72 ± 1.15 ^b^	77 ± 1.15 ^b^	88 ± 1.15 ^b^	93 ± 1.15 ^b^
*Picrorhiza kurroa*	00 ± 0.0 ^a^	00 ± 0.0 ^a^	00 ± 0.0 ^a^	00 ± 0.0 ^a^
**Antimicrobial Potential of Herbal Extracts at Different Concentrations of HCl**				
**Samples**	**Zone of Inhibition (mm)**			
	**0.4% HCl**	**2% HCl**		**4% HCl**
Ciprofloxacin	12.2 ± 0.17 ^b^	15.2 ± 0.17 ^b^		20.2 ± 0.17 ^b^
*Picrorhiza kurroa*	19.8 ± 0.28 ^a^	20.6 ± 0.28 ^a^		23.2 ± 0.23 ^a^
**Antimicrobial Potential of Herbal Extracts at Different a_w_**				
**Samples**	**Zone of Inhibition (mm)**			
	**0.99 a_w_**	**0.95 a_w_**	**0.85 a_w_**	**0.75 a_w_**
Ciprofloxacin	12.4 ± 0.11 ^b^	15.2 ± 0.23 ^b^	18.3 ± 0.11 ^b^	20.2 ± 0.17 ^b^
*Picrorhiza kurroa*	15.4 ± 0.28 ^a^	18.3 ± 0.23 ^a^	20.3 ± 0.28 ^a^	21.4 ± 0.17 ^a^

The means of the triplicate readings were determined, ±SD (standard deviation). Values having different superscripts (^a^, ^b^, and ^c^) in a column show a significant (*p* < 0.05) difference; a_w_: water activity; CFU/mL: colony–forming units per milliliter.

**Table 3:** Antimicrobial potential of picroside–1 compounds against *Yersinia enterocolitica*.

Ethanolic Extracts	Zone of Inhibition (mm)	Activity Index (in Comparison to Ciprofloxacin)
Ciprofloxacin	20.4 ± 0.28 ^a^	-
Picroside–1	23.3 ± 0.34 ^b^	1.14

Data are presented as mean ± SD (*n* = 3); ^a^, ^b^—values having different superscripts in a column showed a significant (*p* < 0.05) difference.

**Table 4:** Ramachandran plot statistics for the DHFR of *Yersinia enterocolitica*.

Most favored regions	[A,B,L]	127	95.5%
Additional allowed regions	[a,b,l,p]	6	4.5%
Generously allowed regions	[~a,~b,~l,~p]	0	0.0%
Disallowed regions	[XX]	0	0.0%

DHFR: Dihydrofolate reductase.

**Table 5:** Total estimated energy and the hydrophilic and hydrophobic interaction between the protein and ligand.

Compound	*Yersinia enterocolitica*		
	E–Total	Hydrophilic Interaction	Hydrophobic Interaction
Picroside–1	−303.24	Trp–22, Gly–26, Glu–27 and Gln–28	Ile–20, Ser–23, Ala–24 and Lys–25

## Data Availability

Not applicable.
